# A transformer-based approach empowered by a self-attention technique for semantic segmentation in remote sensing

**DOI:** 10.1016/j.heliyon.2024.e29396

**Published:** 2024-04-12

**Authors:** Wadii Boulila, Hamza Ghandorh, Sharjeel Masood, Ayyub Alzahem, Anis Koubaa, Fawad Ahmed, Zahid Khan, Jawad Ahmad

**Affiliations:** a Robotics and Internet-of-Things Laboratory, Prince Sultan University, Riyadh 12435, Saudi Arabia; b RIADI Laboratory, National School of Computer Science, University of Manouba, Manouba 2010, Tunisia; c College of Computer Science and Engineering, Taibah University, Medina 42353, Saudi Arabia; d Department of IT and Energy Convergence, Korea National University of Transportation, Chungju, South Korea; e Department of Cyber Security, Pakistan Navy Engineering College, NUST, Islamabad 75350, Pakistan; f School of Computing, Engineering and the Built Environment, Edinburgh Napier University, Edinburgh EH10 5DT, United Kingdom

**Keywords:** Semantic segmentation, Self-attention, Vision transformer, Satellite images, Remote sensing

## Abstract

Semantic segmentation of Remote Sensing (RS) images involves the classification of each pixel in a satellite image into distinct and non-overlapping regions or segments. This task is crucial in various domains, including land cover classification, autonomous driving, and scene understanding. While deep learning has shown promising results, there is limited research that specifically addresses the challenge of processing fine details in RS images while also considering the high computational demands. To tackle this issue, we propose a novel approach that combines convolutional and transformer architectures. Our design incorporates convolutional layers with a low receptive field to generate fine-grained feature maps for small objects in very high-resolution images. On the other hand, transformer blocks are utilized to capture contextual information from the input. By leveraging convolution and self-attention in this manner, we reduce the need for extensive downsampling and enable the network to work with full-resolution features, which is particularly beneficial for handling small objects. Additionally, our approach eliminates the requirement for vast datasets, which is often necessary for purely transformer-based networks. In our experimental results, we demonstrate the effectiveness of our method in generating local and contextual features using convolutional and transformer layers, respectively. Our approach achieves a mean dice score of 80.41%, outperforming other well-known techniques such as UNet, Fully-Connected Network (FCN), Pyramid Scene Parsing Network (PSP Net), and the recent Convolutional vision Transformer (CvT) model, which achieved mean dice scores of 78.57%, 74.57%, 73.45%, and 62.97% respectively, under the same training conditions and using the same training dataset.

## 1 Introduction

Semantic segmentation plays a pivotal role in numerous Remote Sensing (RS) applications [1], [2], [3]. This process involves assigning contextual labels to each pixel in an image, enabling a granular and comprehensive analysis of the scene. In the field of RS, the semantic segmentation of multi-spectral images is especially critical for detailed analysis and data extraction from RS images. This technique is instrumental in identifying various land cover types and areas of interest, as it classifies individual pixels, providing a thorough understanding of the spatial arrangement and scope of different elements within the scene [4].

Semantic segmentation is extensively used in RS for purposes such as mapping land cover, analyzing vegetation, monitoring urban expansion, evaluating disasters, and conducting environmental surveys [5], [6], [7]. It extracts valuable insights from RS data, offering a more nuanced and detailed perspective than traditional classification methods. Deep learning (DL) models, particularly convolutional neural networks (CNNs), have demonstrated exceptional effectiveness in semantic segmentation tasks, learning to distinguish between different classes and objects using extensive training data. The proliferation of high-resolution satellite imagery and annotated datasets has significantly propelled the development and implementation of DL-driven semantic segmentation in RS [8].

Despite remarkable advancements in this field, semantic segmentation still faces several key challenges [9], [10], [11]. One major challenge is understanding and interpreting the spatial connections and context among objects of varying scales [12], [13]. Current models often struggle to grasp the global context of a scene, leading to segmentation errors or incomplete analyses. The size variability of objects in RS images, ranging from expansive structures to smaller entities like vehicles or trees, poses another significant challenge. This size disparity often hampers the accuracy of DL models.

Furthermore, RS images often contain complex and varied backgrounds, including terrain, vegetation, and atmospheric conditions. Implementing attention mechanisms in DL models for RS image segmentation is essential to tackle these issues [14]. The transformative power of transformer-based methods, known for their global modeling capabilities, is gaining traction in RS tasks [15].

This study introduces a transformer-based solution focusing on self-attention for semantic segmentation in RS. Leveraging vision transformers and attention mechanisms in RS semantic segmentation offers several benefits, including global context capture, spatial dependency recognition, and effective handling of intricate backgrounds. Our approach enhances the segmentation accuracy in RS by incorporating transformers and attention mechanisms, thereby addressing global context, diverse object sizes, spatial interactions, and contextual comprehension.

The principal contributions of this study are as follows:

• A vision transformer-based approach, fortified with a self-attention mechanism, is proposed for high-resolution RS image semantic segmentation.
• The proposed methodology, integrating vision transformers and self-attention, effectively captures long-range dependencies and facilitates contextual understanding, thus allowing the model to perceive global context and spatial relations for precise segmentation in complex scenes.
• Inspired by the Swin transformer architecture, images are segmented into several non-overlapping regions instead of working on the entire image. Then, self-attention was applied to these specific regions. This strategy simplifies model optimization and reduces the need for extensive datasets.
• Various experiments were conducted to validate the efficiency of our proposed transformer-attention-based approach in RS semantic segmentation.

The remainder of this paper is organized as follows: Section 2 delves into related research in the domain. Section 3 introduces foundational concepts. Section 4 details our proposed methodology. Section 5 discusses our training methodology and the outcomes. Section 6 underscores the significance of each component of our model via an ablation study. Finally, Sections 7 and 8 present the discussion and conclusions, respectively.

## 2 Related work

In literature, several works have focused on semantic segmentation for RS. In [16], dilated convolutional layers without any downsampling were used to segment small objects from RS images. In [6], a technique that encodes features in two stages was introduced by the authors. In the first stage, a low receptive field was used to segment smaller objects, while in the second stage, downsampling was employed to increase the receptive field, facilitating the segmentation of larger objects. There have also been researches like Multi-Feature Network (MFNet) [17] introducing a multi-feature learning algorithm, incorporating high-level, low-level features and class discriminative features, reducing the confusion between the classes. The authors in [18] have also presented a technique to reduce inter-class confusion. A three-stage mechanism was presented, featuring Image Block Segmentation (IBS) and Superpixel Cluster (SPC) as its main components. These components serve as pre-processing and post-processing algorithms, respectively.

The field of computer vision has been dominated by CNNs for a long time. Remarkable results have been shown by them in all vision tasks, such as image segmentation [19], object tracking [20], object detection [21], and classification.

Self-attention-based transformers gained quick popularity in Natural Language Processing (NLP) when they were introduced by Vaswani et al. [22]. Bidirectional Encoder Representations from Transformers (BERT) [23], which only uses an encoder from the transformer, is trained in two stages called pre-training and fine-tuning. During pre-training, the network is trained on unlabeled data, and then the weights are fine-tuned for any specific task. Popularity has been gained by transformers because they can read the entire sequence of words at once and create a relation between each word. The success of self-attention mechanisms in NLP have inspired their use in computer vision tasks [24], [25], [26].

A pure transformer was directly applied to patches of images by Dosovitskiy et al. [27], achieving excellent results in comparison to state-of-the-art convolutional networks. It was demonstrated that vision transformers are enabled by self-attention to integrate information across the entire image, even in lower layers. Transformers were utilized as the backbone of their network by Ranftl et al. [28] in place of convolutional layers at multiple resolutions, setting new state-of-the-art performance in both monocular depth estimation and segmentation tasks.

In [29], the single-head and multi-head attention mechanisms were compared by Liu et al., with the suggestion that multi-head attention mechanisms result in much more stable training performance.

A novel unsupervised methodology for the efficient analysis and precise segmentation of RS images was introduced in [30]. Segmentation is conducted through a three-step process in this methodology. In the initial stage, the images were divided into equisized blocks. The mean values of the red, green, and blue components of the pixels within each block are computed, forming a feature vector. These feature vectors have a basic clustering algorithm applied to them to achieve a preliminary segmentation result. In the subsequent stage, a Bayesian approach was employed to enhance the preliminary segmentation outcome. Finally, a graph-based technique was employed in the third stage to identify regions with intricate texture structures.

A structure for accomplishing semantic segmentation of aerial images using incomplete annotations was suggested by Hua et al. [31]. The authors suggested labeling a small number of pixels with easy-to-draw scribbles. These annotations were utilized effectively through the proposed FEature and Spatial relaTional regulArization (FESTA) approach. Complementing supervised learning with an unsupervised signal, this technique has considered spatial and feature-related neighborhood structures.

In [32], an approach called Convolutional Vision Transformer (CvT) was introduced. The performance and efficiency of the existing Vision Transformer (ViT) were enhanced by incorporating convolutional elements, thereby amalgamating the strengths of both designs. This advancement was achieved via two principal adjustments: the integration of a novel convolutional token embedding within a hierarchy of Transformers and the utilization of a convolutional projection within a convolutional Transformer block.

A refined deep CNN was introduced in [33]. Building upon HRNet and PSPNet, this network has been engineered to achieve improved segmentation results, thus enabling profound scene analysis and elevating the quality of pixel-level semantic segmentation in high-resolution remote sensing images. The approach primarily revolves around multiband segmentation, employing a foundation of hierarchical multiscale segmentation research. Rule sets for the experimental region's vegetation, buildings, roads, waters, and bare land were established. From this foundation, classification was extracted, and each pixel within the image was labeled with a corresponding category. Leveraging the structure of an image classification network allowed for the utilization of diverse levels of feature vectors to satisfy the classification requirements. The HRNet and PSPNet algorithms were employed for scene analysis, facilitating the acquisition of category labels for all pixels within an image.

A concept called Conv-PVT (Combination of Convolution Blocks and Pyramid Vision Transformer) was introduced in [34] to enhance the overall effectiveness of vision transformers. The authors integrated simple convolution blocks at the initial layer to minimize memory usage through input down-sampling. Comprehensive experiments had been conducted across various tasks, such as image classification, object detection, and segmentation using datasets like ImageNet-1k, COCO, and ADE20k. These experiments assessed the model's accuracy, training time, memory consumption, and resilience. The outcomes indicated that Conv-PVT performed on par with the original PVT while surpassing the performance of ResNet and ResNetXt in certain downstream vision tasks. Moreover, it accomplished this while reducing training time by 60% and diminishing GPU memory usage by 42%. Additionally, Conv-PVT was found to have achieved twice the inference speed of PVT5.

Numerous approaches and techniques have been developed in the field of semantic segmentation, yet our proposed method establishes a unique position among the available solutions. Here, we highlight the key distinctions and improvements our approach provides relative to the discussed works:

• **Resolution and Receptive Field Trade-off**: The importance of details in RS is signified by the emphasis on high resolution, as shown by approaches like [16] and the two-stage encoding method proposed by [6]. Similarly, the self-attention mechanism is leveraged in our work, ensuring that both resolution and object segmentation are treated with paramount importance, thereby providing an optimal balance.
• **Unsupervised Learning and Bayesian Refinement**: The potential of unsupervised strategies is underscored by the methodology proposed by Song & Qu [30], which involves clustering feature vectors and subsequently refining them through Bayesian techniques. Such unsupervised signals could be integrated into our primarily supervised method, potentially augmenting our segmentation outcomes with additional robustness, especially in scenarios of data scarcity.
• **Sparse Annotations and Neighborhood Structures**: The work of Hua et al. [31] revolves around the exploitation of sparse annotations, with an emphasis on neighborhood structures in both the spatial and feature domains. This could parallel the focus of our approach to capturing spatial context. By potentially incorporating such scribbled annotations, the adaptability of our model in real-world scenarios might be further enhanced.
• **Feature Learning and Confusion Reduction**: Emphasis on multi-feature learning for inter-class confusion reduction is provided by solutions like MFNet [17]. Upon this, our model builds by introducing an adaptive window-based self-attention, which is provided to yield more distinct and contextually accurate feature representations.
• **Transformers in Vision with Convolutions**: Impressive capabilities have been demonstrated by transformers in vision, as seen in [27] and [28], while newer works like CvT [32] merge convolutions with transformers. This fusion is mirrored by our approach, albeit with innovations tailored specifically for remote sensing, ensuring a more granular attention mechanism is provided.
• **Hierarchical Multiscale Segmentation**: The power of hierarchical multiscale segmentation is tapped into by the fusion of HRNet and PSPNet by Sun & Zheng [33]. Similarly, patterns across multiple scales are aimed to be captured by our model by harnessing the inherent capabilities of transformers, thereby providing richer contextual insights.
• **Efficiency Enhancements in Transformers**: The computational efficiency of transformers is aimed to be enhanced by the Conv-PVT model proposed by Zhang & Zhang [34]. This objective is aligned with our work, which focuses on computational efficiency through the selective application of self-attention and the optimization of computational demands for large-scale RS datasets.

The proposed method integrates features from several leading-edge techniques, tailoring them specifically to overcome the inherent challenges of semantic segmentation in RS. This will help to open new avenues and establish a foundation for further advancements in the field. A detailed comparison of our approach with existing models is provided in Table 1, which highlights the distinctive features, advantages, and potential limitations of each method. This comparative analysis underscores the contributions of our methodology, setting a new benchmark in the field of RS semantic segmentation.

**Table 1:** Summary of existing models.

Ref	DL Method	Main Steps	Advantages	Drawbacks
[17]	Multi-Feature learning algorithm	Utilizes ASSP for contextual features.	Reduces class confusion like trees vs grass.	Struggles with generalization across diverse datasets.
[18]	Three-stage mechanism for class confusion	Superpixel clustering and block segmentation.	Improves boundaries and computational efficiency.	Increased computational requirements, less efficient for real-time applications.
[35]	Multiscale deformable CNN	Light-weight network with dense conditional random fields.	Balanced computational complexity and feature extraction.	Computationally expensive, requires more training data.
[27]	Transformer on image patches	Self-attention on patches across multiple datasets.	Generalized but computationally heavy.	High computational cost, large training data requirement.
[36]	Vision transformer on image windows	Self-attention within windows with cyclic shift.	Reduces computational complexity.	Challenges in capturing very fine details.
[37]	YOLT and SSD for object detection	Resolution enhancement effects on detection performance.	Higher resolution improves mAP.	Struggles with small or overlapping objects, SSD has lower accuracy.
[16]	Full-resolution segmentation	Dilated convolutions replace pooling layers.	Better at segmenting small objects.	Increased computational load, slower inference times.
[30]	Unsupervised segmentation	Three-stage segmentation with Bayesian and graph-based methods.	Efficient and accurate for complex textures.	May not perform well compared to supervised methods with abundant, high-quality data.
[31]	FESTA method for sparse annotations	Combines supervised and unsupervised learning.	Effective use of sparse annotations.	Underperformance in complex scenes with intricate object relationships.
[32]	CvT: Convolutions in ViT	Hierarchy of Transformers with convolutional tokens.	Enhances ViT performance and efficiency.	Complexity in integration, difficulties in optimization and training.
[33]	HRNet + PSPNet for high-res images	Multiband pixel-level segmentation.	Improved pixel-level segmentation.	High computational requirements for high-resolution images.
[34]	Conv-PVT for efficient training	First-layer convolutions for down-sampling.	Faster training, less GPU memory.	Trade-off with depth of feature extraction, affecting performance in some tasks.

## 3 Background

Semantic segmentation, the task of classifying each pixel in an image into a particular class, has become a pivotal computer vision problem with many applications, including medical imaging, autonomous driving, and remote sensing (RS) [38]. The objective is not just to identify the presence of specific features but to delineate the precise boundaries and spatial characteristics of these features within the image.

• **Remote Sensing and its Unique Challenges**: RS images, particularly high-resolution satellite images, pose distinct challenges. The vastness of the captured scenes implies high intra-class variability, numerous small objects, and significant scale variability. Conventional convolutional architectures might lose out on capturing such fine-grained details, especially when downsampling operations reduce the spatial resolution of feature maps.
• **Transformers in Computer Vision**: Initially designed for natural language processing tasks, they have been repurposed for computer vision problems due to their self-attention mechanism [39]. Unlike convolutional layers with a fixed and local receptive field, the self-attention mechanism can capture long-range dependencies and contextual information.
• **Self-Attention Mechanism**: The crux of the self-attention mechanism lies in its ability to compute a weighted combination of all input features based on their relevance. It utilizes three primary components – Query, Key, and Value – to derive these weights. The weight between any two pixels, say *i* and *j*, in the image, is computed as a function of their corresponding Key and Query representations. This allows the model to focus on relevant parts of the image, regardless of their spatial proximity.
• **CNNs in Remote Sensing**: Traditional CNNs, while effective for many vision tasks, leverage pooling layers to gather context information, inevitably reducing the spatial resolution. This trade-off between spatial resolution and receptive field size is detrimental for RS, where the segmentation of smaller objects with high precision is of utmost importance [40], [41].

In the subsequent sections, we delve into a novel approach that seeks to amalgamate the strengths of transformers and CNNs, aiming to address the unique challenges posed by RS images and enhance the granularity and accuracy of semantic segmentation [42].

## 4 Proposed method

This section delves deep into our proposed network architecture, elucidating both the rationale behind our design choices and the mathematical foundations that underpin its performance.

Central to our technique is the information retrieval capability of the key, query, and value mechanism. This mechanism is renowned for its ability to achieve a global receptive field. Transformer-based techniques, especially those employing self-attention, are powerful and generalized systems. However, they often demand vast amounts of data. This becomes a bottleneck for RS applications where datasets are inherently limited. The challenge arises from the nature of the self-attention mechanism: each output is intricately linked to all input values, making optimization a herculean task as the input size grows.

The convolutional operation is a linchpin in our approach, serving as the primary mechanism for spatial feature extraction. As a filter or kernel traverses the input feature map, it computes an output at each position. This output is derived from element-wise multiplication with the overlaid image segment, followed by summation. These adaptive filters, which undergo refinement during training, excel at discerning spatial patterns, ranging from edges to intricate textures. Their proficiency is contingent on their depth within the network. This operation is mathematically captured in Equation (1):

(1) $$O(x,y)=\sum_{i=-\infty }^{\infty }\sum_{j=-\infty }^{\infty }I(i,j)\cdot K(x-i,y-j)$$

Here, *O* represents the output feature map, *I* the input feature map, and *K* the kernel or filter.

**Figure 1:**
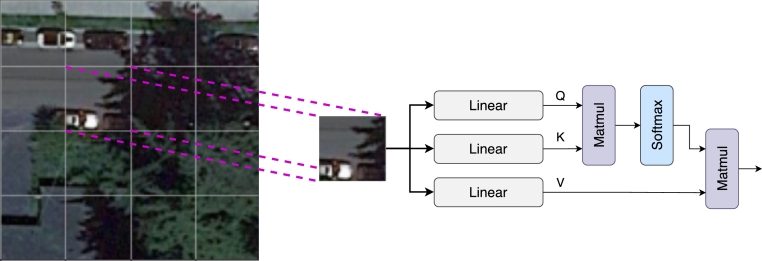
Illustration of the self-attention mechanism applied per region.

Segmentation accuracy is significantly enhanced by assimilating information from neighboring pixels. However, indiscriminate aggregation from the entire image can be counterproductive, potentially decelerating optimization. Addressing this, we segment feature maps into fixed, non-overlapping regions. For each region, the Key, Query, and Value are computed, forming the bedrock for self-attention, as illustrated in Fig. 1. The self-attention mechanism is encapsulated by Equation (2):

(2) $$Attention(Q,K,V)=Softmax(\frac{QK^{T}}{\sqrt{d_{k}}})V$$

In this equation, $\sqrt{d_{k}}$ acts as a scaling factor, with $d_{k}$ denoting the dimensionality of the keys and values. The matrices *Q*, *K*, and *V* emerge from applying a Linear layer to input features, ensuring that the attention weights are appropriately scaled.

**Figure 2:**
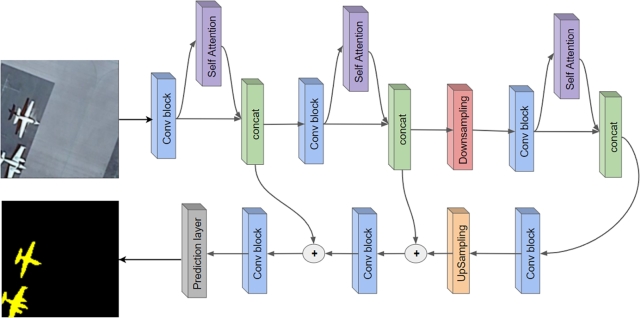
Overview of the proposed methodology's architecture.

High-resolution RS imagery presents a diverse landscape of objects, varying in size and necessitating different receptive fields. Our encoder, depicted in Fig. 2, processes features at full resolution, effectively segmenting smaller objects. Concurrently, the self-attention mechanism collates essential contextual information, providing a holistic view of the scene. Each convolutional block is a composite of six convolution layers, succeeded by a 1x1 kernel convolution layer. This layer plays a pivotal role, halving the channel count before feeding into the self-attention block. The block houses two self-attention layers, with the second layer undergoing a cyclic shift, a technique inspired by the Swin Transformer. This shift facilitates information sharing between windows, bolstering model performance. Features from self-attention and convolution blocks are concatenated, ensuring a rich feature representation. The convolutional block, enriched with both local and global features at each pixel, is empowered with a wealth of information. The subsequent 1x1 convolutional layer discerns the most pertinent information at each pixel by halving the channels. Downsampling employs a 2D convolutional layer with a 3x3 kernel, a stride of 2, and padding of 1, reducing spatial resolution by half. Upsampling leverages bilinear interpolation, complemented by two 3x3 kernel convolutional layers.

Algorithm 1 offers an overview of our technique's inference pipeline, with various feature map notations elucidating the transformation stages.

**Algorithm 1:**
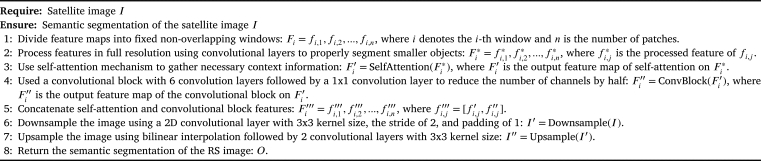
Transformer-based approach with self-attention for semantic segmentation in remote sensing.

## 5 Experimentation and results

We have used the Dota dataset [43] for the experimentation. It contains 1411 images with their segmentation masks. Each image was cropped into 512x512 patches along with their segmentation masks. These patches were then saved and used as training data. We had 8,972 samples, from which we separated them with 8:2 ratio.

The dataset is highly imbalanced; therefore, while splitting data into train and validation sets, we ensured that each class was represented in each dataset at equal proportions. To prevent over-fitting during training, the input samples are augmented using horizontal and vertical flips at random.

We used Adam as our optimizer with a learning rate of 0.001 and momentum of 0.9. Cross-entropy loss, along with dice loss, was used to train our network over 100 epochs. Other hyperparameters, such as dropout rate and weight decay, were set to *X* and *Y*, respectively, to prevent overfitting and ensure model convergence. We also utilized batch normalization after each convolutional layer to stabilize learning and accelerate the training process. The training was performed with a batch size of *Z*, striking a balance between computational efficiency and memory usage.

### 5.1 Dataset description

The DOTA (Dataset for Object Detection in Aerial Images) encompasses a diverse and extensive collection of aerial photographs, recognized for their variety and complexity. This makes it a prime benchmark for testing both object detection and semantic segmentation methodologies within the scope of RS imagery [43].

#### 5.1.1 Dataset size and diversity

Comprising more than 2,800 aerial photographs, the DOTA dataset's image dimensions range from roughly 800x800 to 4,000x4,000 pixels. These high-resolution photographs capture a broad spectrum of scenes, from natural landscapes to urban environments, featuring diverse objects like vehicles, buildings, ships, airplanes, and athletic fields. The dataset covers a wide range of environmental conditions and lighting variations, offering a rich and varied collection for thorough assessment.

#### 5.1.2 Annotations and object categories

One of the significant attributes of the DOTA dataset is its comprehensive annotation system. Each photograph within the dataset is accurately marked with oriented bounding boxes, providing precise details on the position and orientation of various objects. It includes 15 distinct object categories, each with variations in size, shape, and orientation, contributing to the dataset's intricacy and the challenge of precise object detection.

#### 5.1.3 Challenges posed by the dataset

The DOTA dataset introduces several common challenges associated with aerial image analysis, notably:

• **Scale Variability**: The dataset features objects of varying sizes, requiring algorithms that are capable of accurately detecting objects of both large and small dimensions.
• **High Object Density**: Certain images in the dataset display a high density of objects, necessitating algorithms that can effectively differentiate between objects in close proximity.
• **Diverse Backgrounds**: The wide array of landscapes and urban settings demands robust feature extraction techniques to manage the complexity of various backgrounds.

#### 5.1.4 Suitability for evaluating generalizability

The diversity and intricacy of the DOTA dataset render it an excellent tool for gauging the generalizability of object detection and segmentation methods. Its array of object types and challenging scenarios serve as a rigorous testing ground to evaluate the efficacy of different algorithms under diverse conditions.

In relation to our proposed approach, the DOTA dataset, with its varied contents and inherent challenges, validates the adaptability and robustness of our model, affirming its efficiency without the necessity for extensive datasets. The dataset effectively encapsulates the complexities of real-world scenarios, enabling a comprehensive evaluation of our method's performance and its general applicability.

### 5.2 Computational resource analysis

This section delves into the computational demands and processing times required by our proposed model throughout its training phase, offering a quantitative review of its efficiency across 100 epochs. This analysis is essential for understanding the practical deployment of the model in scenarios where computational resources might be limited.

We conducted our experiments on a system equipped with an RTX 3060 12 Gb GPU and a Core i7 12th generation CPU. The batch size was maintained at 8, and we used an image resolution of 128x128 pixels. This setup ensured that we did not fully exploit the GPU's maximum capacity, thus allowing us to evaluate the model's performance without pushing the hardware to its limits.

Throughout 100 epochs, a consistent observation was made regarding GPU memory usage: 45.65 MB was allocated, and 1668.00 MB was cached for each epoch. The stability of these GPU memory metrics is notable, highlighting the efficiency of our model in terms of memory usage. Despite the introduction of additional computational elements in our architectural design, the minimal rise in memory allocation indicates our effective architectural strategies, which prevent excessive memory consumption. This aspect is particularly beneficial when scaling up to handle larger datasets or images with higher resolutions.

### 5.3 Evaluation matrix

The assessment of segmentation models requires diverse metrics, each shedding light on different aspects of a model's precision and efficacy. We have selected a comprehensive array of evaluation metrics for our model, including dice score, intersection over union [IOU], precision, recall, specificity, and accuracy. Each metric offers a distinct perspective on the model's capabilities.

*Dice score*  Also known as the Sørensen-Dice coefficient, the Dice Score measures the degree of similarity between two binary samples. In segmentation contexts, it assesses how closely the predicted mask aligns with the actual ground truth. A higher Dice Score indicates a closer resemblance between the model's predictions and the real data.

*Intersection over union (IoU)*  This metric, similar in concept to the Dice Score but differing in its mathematical formulation, evaluates the overlap between two binary samples. A model achieving a higher IoU indicates a more significant overlap between its predictions and actual segmentations.

*Precision*  Precision assesses the accuracy of positive predictions made by the model. It evaluates the proportion of true positive samples among all positive predictions. A model with high precision suggests fewer false positives and greater reliability in detected objects within the segmentation task.

*Recall*  Also known as sensitivity, recall measures the model's ability to correctly identify true positives. In segmentation, it reflects the model's capacity to detect all relevant objects or regions. A higher recall implies fewer true positives being missed.

*Specificity*  This metric focuses on the correct identification of negatives, complementing recall. For segmentation, it means accurately excluding areas that are not of interest.

*Accuracy*  Accuracy provides a comprehensive view of the model's performance, considering both positive and negative predictions. It reflects the frequency of the model's predictions aligning with the truth across the entire dataset.

*Average precision (AP)*  AP, calculated at different IoU thresholds, offers a balance between precision and recall for specific overlap criteria between predicted and actual masks. Higher AP values at more stringent thresholds indicate the model's precision and strength in segmentation tasks.

*Mean average precision (mAP)*  This metric represents the average of AP values across different IoU thresholds, providing a singular metric that encapsulates the model's overall precision in segmentation. A higher mAP indicates strong performance in accurately predicting segmentation masks across various levels of overlap stringency.

Table 2, Table 3 provide a comparative analysis of various segmentation models using the metrics discussed. Our proposed network, when benchmarked against other popular architectures, demonstrates a commendable performance, highlighting its efficacy in addressing segmentation challenges.

**Table 2:** Comparison of the segmentation accuracy.

Network	mIoU	Dice Score	Precision	Recall	Specificity	Accuracy
PSP Net	64.89	73.45	68.84	75.97	99.41	98.67
FCN	65.72	74.57	59.14	69.73	98.53	96.26
UNet	71.62	78.57	80.31	85.13	99.66	99.26
CvT	54.11	62.97	67.93	70.37	99.47	98.60

**Proposed Network**	**73.57**	**80.41**	**81.36**	**85.83**	**99.67**	**99.30**

**Table 3:** Comparison of the segmentation accuracy.

Network	AP65	AP75	AP80	AP90	mAP
PSP Net	87.75	83.60	80.64	70.14	80.53
FCN	91.79	87.62	83.92	64.40	81.93
UNet	**92.72**	87.92	83.39	68.07	83.02
CvT	90.49	90.15	89.97	89.37	89.99

**Proposed Network**	91.19	**91.03**	**90.91**	**90.62**	**90.93**

**Figure 3:**
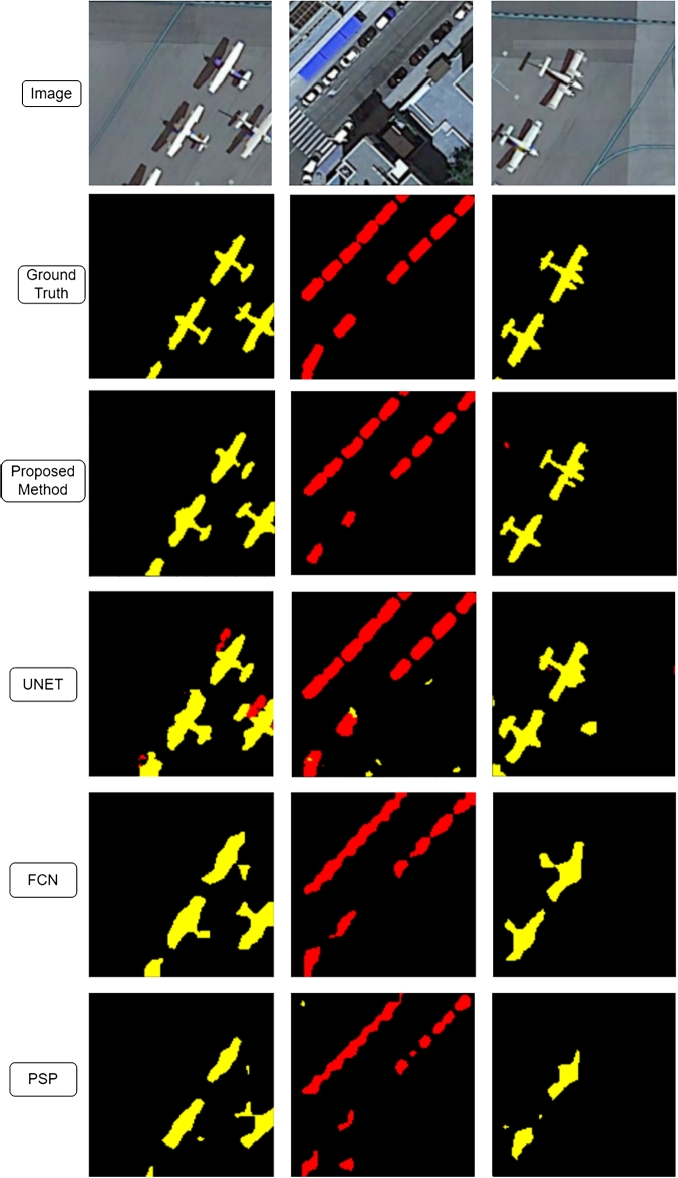
Semantic segmentation results achieved by our proposed network, UNet, FCN, and PSP.

Fig. 3 shows the semantic segmentation results achieved by our proposed network, UNet, FCN, and PSP Net, illustrating the practical application and visual performance of these models in comparison.

### 5.4 Early training performance evaluation

#### 5.4.1 Validation metrics improvement

Following the 100-epoch training regimen on the Dota dataset, we observed notable enhancements in the model's validation performance. By the 10th epoch, validation IoU achieved 44.9%, and validation Dice reached 54.24%, signifying the model's enhanced precision in segmenting images accurately.

Validation accuracy peaked at 97.63%, indicating the model's robust overall performance on the validation set. This was further supported by validation precision, which stood at 56.69%, denoting a substantial rate of true positives amongst the predicted positives.

Moreover, validation recall and validation specificity were recorded at 54.48% and 98.85%, respectively, underscoring the model's efficacy in correctly identifying both positive and negative samples—an essential aspect, particularly in imbalanced datasets.

#### 5.4.2 Analysis of average precision at IoU thresholds

The AP values quantified our model's precision across different IoU thresholds, which offer a detailed view of its precision and recall. At the 65% IoU threshold (AP65), the model registered an AP of 74.7%, and it remained resilient at higher thresholds with AP75, AP80, and AP90 at 73.93%, 73.48%, and 72.02%, respectively. The mAP across these varied thresholds stood strong at 73.53%, indicative of the model's consistent accuracy in segmentation.

#### 5.4.3 Visualization of validation metrics

**Figure 4:**
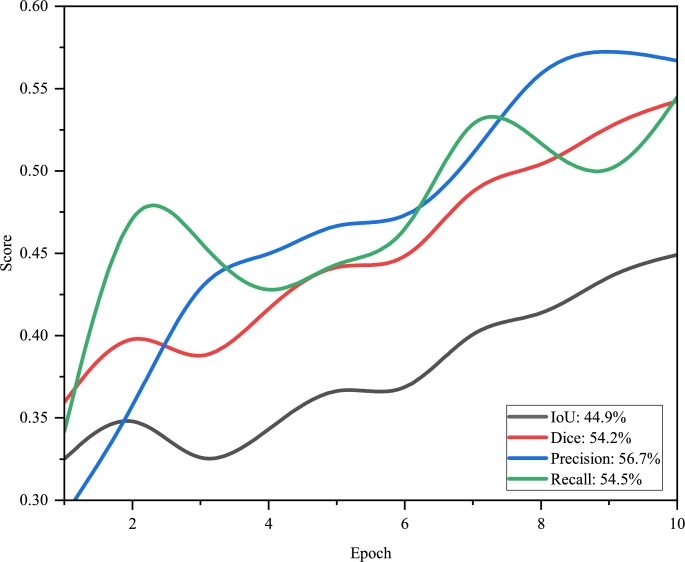
The distribution of validation metrics over the first 10 epochs.

**Figure 5:**
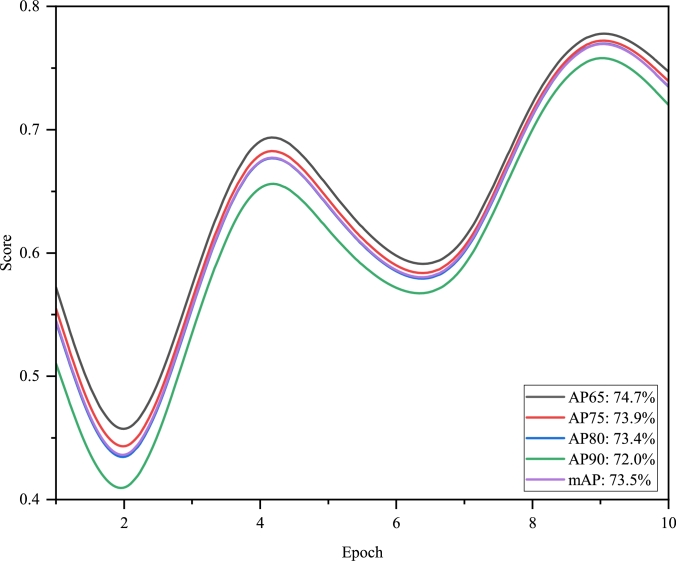
AP metric progression over initial 10 epochs for different IoU thresholds and mAP.

**Figure 6:**
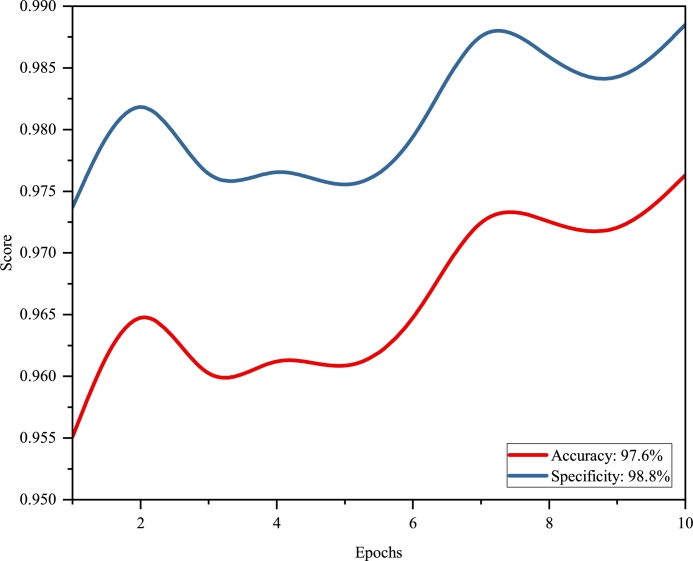
Validation accuracy and specificity evolution over first 10 epochs.

To clearly depict the progression of the model's validation performance, we propose dividing the visualization into three separate figures: 4, 5, and 6. Fig. 4 illustrates the distribution lines for Validation IoU, Validation Dice, Validation Precision, and Validation Recall. Fig. 5 focuses on the distribution of AP65, AP75, AP80, AP90, and mAP. Lastly, Fig. 6 presents Validation Accuracy and Validation Specificity, providing a comprehensive view of the model's performance.

### 5.5 Quantitative evaluation of model consistency

Several experiments were conducted to ascertain the dependability and uniformity of our model. We analyzed both the average and standard deviation of critical performance indicators at different training stages, encompassing Average Precision (AP) at various IoU levels and vital validation metrics at the ${\text{10}}^{\text{th}}$, ${\text{50}}^{\text{th}}$, and ${\text{100}}^{\text{th}}$ epochs.

Results in Table 4 offer a comprehensive view of the model's performance consistency encapsulated at these distinct epochs.

**Table 4:** Model performance consistency across four runs over various epochs.

Metric	Epoch 10		Epoch 50		Epoch 100	
	Mean	Std	Mean	Std	Mean	Std
AP65	0.6643	0.0923	0.8529	0.0360	0.8833	0.0379
AP75	0.6590	0.0915	0.8497	0.0374	0.8809	0.0381
AP80	0.6560	0.0908	0.8480	0.0376	0.8794	0.0385
AP90	0.6457	0.0902	0.8431	0.0395	0.8760	0.0387
mAP	0.6563	0.0912	0.8484	0.0377	0.8799	0.0383
IOU	0.4418	0.0151	0.4966	0.0257	0.5463	0.0334
Dice	0.5340	0.0163	0.5888	0.0271	0.6411	0.0311
Accuracy	0.9773	0.0022	0.9825	0.0014	0.9849	0.0012
Precision	0.5240	0.0451	0.6192	0.0276	0.6173	0.0218
Recall	0.5586	0.0335	0.6794	0.0256	0.7435	0.0204
Specificity	0.9907	0.0028	0.9935	0.0012	0.9959	0.0007

The detailed examination of our model's performance at epochs 10, 50, and 100, as illustrated in Table 4, reveals significant insights into its developmental trajectory and stability during training. From this data, several key insights emerge.

Firstly, there is a noticeable trajectory of performance enhancement from the ${\text{10}}^{\text{th}}$ to the ${\text{100}}^{\text{th}}$ epoch, as reflected by an increase of mean values in mAP, Validation IOU, Dice Score, and other metrics. This improvement signifies the model's growing proficiency in delivering accurate and precise segmentation as training progresses. The increase in mAP, in particular, demonstrates the model's enhanced ability to discriminate between classes accurately.

However, an interesting observation was the high accuracy achieved by the proposed model within the first few epochs of training, which did not significantly increase in subsequent iterations. This phenomenon can be primarily attributed to the quality and simplicity of the DOTA dataset, which facilitated rapid initial learning. The dataset's well-annotated and clear imagery allowed our model to quickly assimilate the fundamental features necessary for accurate object detection and segmentation of aerial images.

Despite achieving high accuracy in early epochs, we continued the training for an extended number of epochs. The purpose was to refine the model's understanding of more nuanced aspects of the data and to enhance its generalization capabilities, especially for subtle and complex scenarios not immediately captured in the early training phase. Training our model on additional epochs contributed more towards enhancing the model's robustness and its ability to handle a variety of complex segmentation tasks. Moreover, the declining standard deviations over epochs in various metrics further indicate a reduction in performance variability, thereby enhancing the model's predictability and reliability across different scenarios. This consistency is crucial in RS applications where varied conditions and object presentations are common. In addition, the consistent improvement in both Precision and Recall metrics throughout the training epochs highlights the model's increasing accuracy in correctly identifying positive cases (Precision) while minimizing false negatives (Recall). This balance is essential in scenarios where the cost of missing true positives can be high. It is also noteworthy that the Specificity metric remains high throughout the training process, reaffirming the model's ability to accurately identify true negatives. This is particularly important in datasets with a large number of negative samples, such as the DOTA dataset used in our study.

As we move forward, it is imperative to consider how these findings can be translated into practical applications in RS. The ability of our model to effectively segment objects in high-resolution aerial images opens up avenues for its application in various fields such as urban planning, agricultural monitoring, and environmental conservation. Its robust performance across diverse conditions suggests that it can be a reliable tool in these domains.

Future research could focus on further improving the model's performance with more diverse and challenging datasets. Experimentation with different architectures or hybrid models combining the strengths of various techniques could also yield fruitful results. Additionally, exploring dynamic windowing techniques, as mentioned earlier, could provide a more adaptive approach to handling varying object sizes and complexities in images.

## 6 Ablation study

To understand the impact of each architectural component on our results, we conducted a series of experiments by modifying parts of the model and analyzing the outcomes of each variant.

### 6.1 Without self-attention

In this variant, we removed the self-attention mechanisms from our architecture. This modification was intended to assess the role of self-attention in capturing context information necessary for effective segmentation.

**Figure 7:**
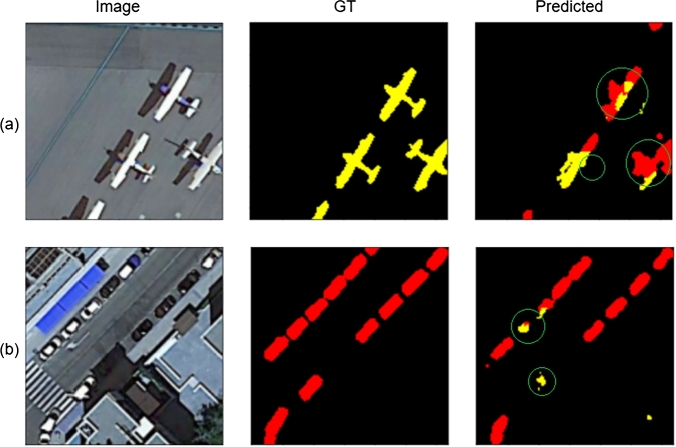
Results achieved without self-attention.

The results, as illustrated in Fig. 7, reveal a reduced capacity for segmenting detailed features such as the tail of the airplane and distinguishing between the plane and its shadow (see Fig. 7(a)). Similarly, in tasks where the objective is to identify cars (small objects) adjacent to buildings (large objects), as shown in Fig. 7(b), there is a noticeable challenge. These observations highlight the significance of self-attention mechanisms in managing complex segmentation tasks. In Fig. 7, ‘GT’ refers to the ground truth, and ‘Predicted’ refers to the semantic segmentation performed without the self-attention mechanism.

### 6.2 With self-attention

Contrasting the previous setup, we applied the self-attention mechanism across the entire image without dividing the features into non-overlapping areas. This experiment aimed to evaluate the effectiveness of global versus localized self-attention.

**Figure 8:**
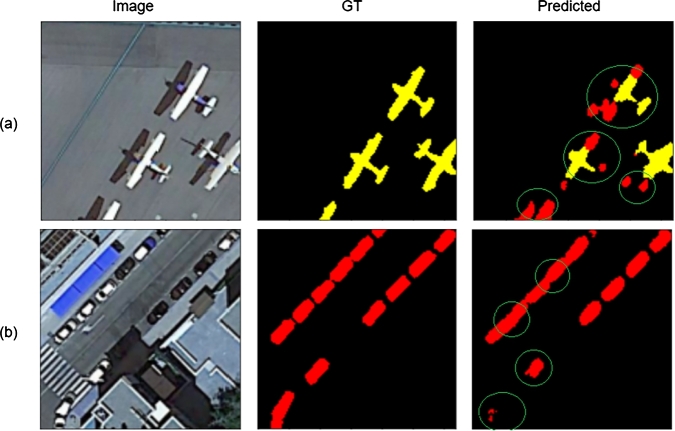
Results achieved by applying self-attention to the whole image.

As seen in Fig. 8(a,b), while there is an improvement in large object segmentation, the model still struggles with accurately differentiating between closely situated classes.

### 6.3 Performance comparison

We conducted a performance comparison between the model variants with and without self-attention. The model without self-attention achieved a validation mIoU of 42.81%, Dice Score of 52.33%, Precision of 67.02%, Recall of 70.26%, Specificity of 99.47%, and Accuracy of 98.52%, with an mAP of 85.24%. In contrast, the model with self-attention significantly outperformed in all metrics, indicating the efficacy of self-attention in improving segmentation accuracy. This comparative analysis is summarized in Table 5.

**Table 5:** Performance comparison of the model with and without self-attention at epoch 100.

Measure	Without Self-Attention	With Self-Attention
mIoU	42.81	**73.57**
Dice Score	52.33	**80.41**
Precision	67.02	**81.36**
Recall	70.26	**85.83**
Specificity	99.47	**99.67**
Accuracy	98.52	**99.30**
mAP	85.24	**90.93**

### 6.4 Impact of self-attention on computational efficiency

**Figure 9:**
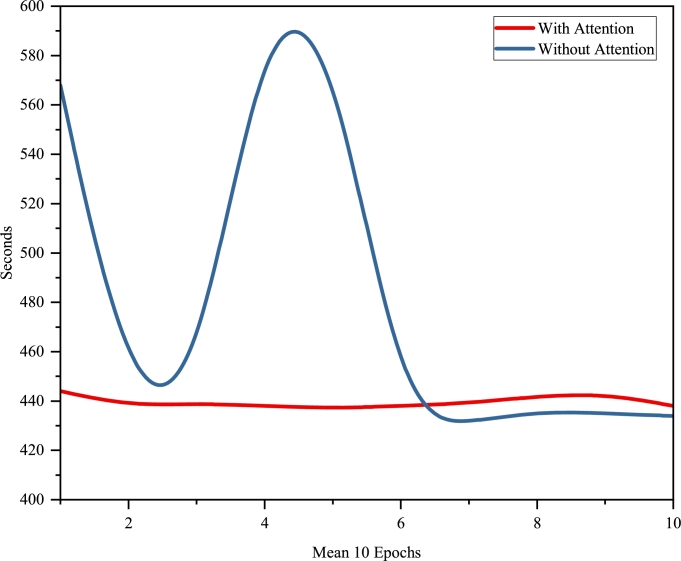
Comparative distribution of average epoch times.

In addition to performance metrics, we analyzed the average epoch time for models with and without self-attention mechanisms. The inclusion of self-attention led to a decrease in average epoch time, enhancing processing efficiency as illustrated in Fig. 9.

**Figure 10:**
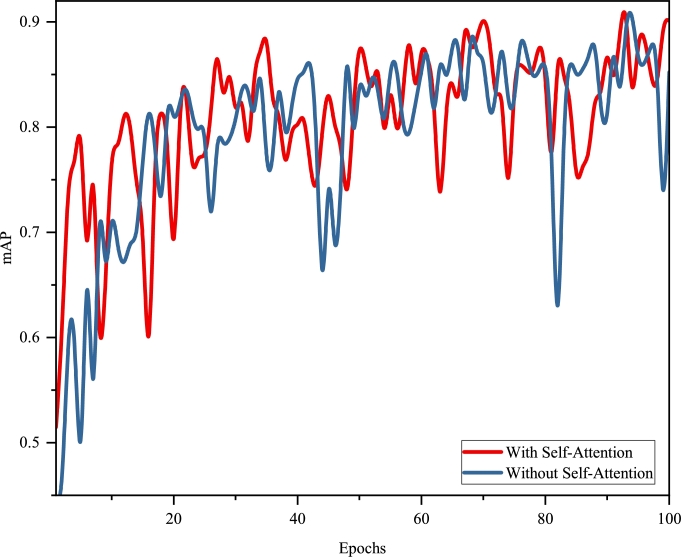
Distribution of mAP over training epochs for models with and without self-attention.

Furthermore, we present a distribution of the mAP over the training epochs for both model variants, visually depicting the performance improvement over time. The distribution is shown in Fig. 10.

These comparative analyses underscore the significant impact of the self-attention mechanism on both the model's segmentation accuracy and computational efficiency.

## 7 Discussion

This study proposes a transformer-based approach for semantic segmentation in RS. This approach utilizes a self-attention mechanism to improve the segmentation of smaller objects by processing features in high resolution. The self-attention mechanism used in this way ensures that the model does not rely on pooling operations for context information.

In this study, semantic segmentation of multi-spectral images is proposed. These images are acquired across a diverse range of electromagnetic spectrum wavelengths. Each band in a multi-spectral image corresponds to a distinct wavelength interval, offering unique insights. This rich, detailed data allows for highly accurate discrimination and classification of various materials and objects, rendering multi-spectral imaging exceedingly advantageous for RS applications, including land cover categorization, vegetation health analysis, and aquatic quality monitoring. The primary advantage of multi-spectral imaging lies in its enhanced classification precision, owing to the extra spectral details that can distinguish features according to their spectral signatures.

In order to gather an adequate amount of context information, a large enough receptive field is necessary for the segmentation or detection of any object. This is normally done by adding multiple pooling layers, which also reduces the spatial resolution of the features [44].

To properly segment small objects, it is imperative that we process the features in high resolution while also maintaining a large enough receptive field [16]. This is where our self-attention mechanism shines; convolutional layers are responsible for processing local information with a low receptive field, while self-attention mechanisms provide the necessary context information. The authors in [6] have used dilated convolution to layers to process local features and then combined them with the global features achieved using downsampling. This is also a good way to solve this issue. However, it requires multiple simultaneous dilated convolution layers to achieve a reasonable receptive field.

Unlike traditional transformer mechanisms [27], our self-attention mechanism does not have a global receptive field. It can be argued that not having a global receptive field might limit the performance of our model. This might be true in applications that work with small input images. However, when working with high-resolution RS images, it is impractical to apply a global receptive field since it would drastically increase the resources needed to run the model. To reduce the computational requirements, we divided our input image into several windows, and then self-attention was applied in each of these windows. This gives a limited amount of surrounding information to each pixel and is easier to optimize. Although using Windows can reduce the need for huge datasets to some extent, increasing the size of the dataset can still improve the generalization of the network and its overall performance.

The amount of context information required is different for each object. In our case, the window size is fixed; therefore, the amount of surrounding information is also fixed. Future works can try to integrate dynamic windowing where the size of the window changes as per the requirement of the task, allowing the model to better understand the spatial relationships between different parts of the image and to focus on the most important features for classification.

## 8 Conclusion

This study proposed a technique that combines convolution and self-attention mechanisms. It shows the importance of dividing the image into windows and applying a self-attention mechanism in each window. This windowing method can reduce computational resources, and unlike traditional transformer networks, it also eliminates the need for large datasets and pretraining of the networks. Without the information gathered by self-attention mechanisms, our model seems to face problems identifying larger objects. Removing the windowing mechanism and applying self-attention to the whole image has been shown to have optimization problems and would also require more computational resources. This approach can be used to identify a wide range of objects in satellite images, such as buildings, roads, water bodies, vegetation, vehicles, and other objects. For future work, we plan to apply our approach to additional datasets in order to comprehensively evaluate and verify its performance across diverse scenarios.

## Data availability statement

The data that support the findings of this study are openly available in https://captain-whu.github.io/DOTA/dataset.html.

## CRediT authorship contribution statement

**Wadii Boulila:** Writing – review & editing, Writing – original draft, Visualization, Validation, Supervision, Project administration, Methodology, Formal analysis, Conceptualization. **Hamza Ghandorh:** Writing – original draft, Supervision, Project administration, Investigation, Funding acquisition. **Sharjeel Masood:** Writing – original draft, Visualization, Validation, Software, Formal analysis, Data curation, Conceptualization. **Ayyub Alzahem:** Writing – review & editing, Writing – original draft, Visualization, Validation, Software, Formal analysis. **Anis Koubaa:** Writing – review & editing, Supervision, Investigation, Formal analysis. **Fawad Ahmed:** Writing – review & editing, Supervision, Project administration, Investigation. **Zahid Khan:** Writing – review & editing, Visualization, Project administration, Investigation. **Jawad Ahmad:** Writing – review & editing, Supervision, Resources, Project administration, Investigation.

## Declaration of Competing Interest

The authors declare that they have no known competing financial interests or personal relationships that could have appeared to influence the work reported in this paper.
